# Inhibition of cyclin-dependent kinase 5 affects early neuroinflammatory signalling in murine model of amyloid beta toxicity

**DOI:** 10.1186/s12974-017-1027-y

**Published:** 2018-01-04

**Authors:** Anna Wilkaniec, Magdalena Gąssowska-Dobrowolska, Marcin Strawski, Agata Adamczyk, Grzegorz A. Czapski

**Affiliations:** 1 Department of Cellular Signalling, Mossakowski Medical Research Centre Polish Academy of Sciences, Pawińskiego 5, 02-106 Warsaw, Poland; 2 Laboratory of Electrochemistry, Faculty of Chemistry, University of Warsaw, Pasteura 1, 02-093 Warsaw, Poland

**Keywords:** Neuroinflammation, Amyloid beta, Alzheimer’s disease, Cdk5, Cytokines, Gene expression

## Abstract

**Background:**

Cyclin-dependent kinase 5 (Cdk5) belongs to the family of proline-directed serine/threonine kinases and plays a critical role in neuronal differentiation, migration, synaptogenesis, plasticity, neurotransmission and apoptosis. The deregulation of Cdk5 activity was observed in post mortem analysis of brain tissue of Alzheimer’s disease (AD) patients, suggesting the involvement of Cdk5 in the pathomechanism of this neurodegenerative disease. However, our recent study demonstrated the important function of Cdk5 in regulating inflammatory reaction.

**Methods:**

Since the role of Cdk5 in regulation of inflammatory signalling in AD is unknown, we investigated the involvement of Cdk5 in neuroinflammation induced by single intracerebroventricular (icv) injection of amyloid beta protein (Aβ) oligomers in mouse. The brain tissue was analysed up to 35 days post injection. Roscovitine (intraperitoneal administration) was used as a potent Cdk5 inhibitor. The experiments were also performed on human neuroblastoma SH-SY5Y as well as mouse BV2 cell lines treated with exogenous oligomeric Aβ.

**Results:**

Our results demonstrated that single injection of Aβ oligomers induces long-lasting activation of microglia and astrocytes in the hippocampus. We observed also profound, early inflammatory response in the mice hippocampus, leading to the significant elevation of pro-inflammatory cytokines expression (e.g. TNF-α, IL-1β, IL-6). Moreover, Aβ oligomers elevated the formation of truncated protein p25 in mouse hippocampus and induced overactivation of Cdk5 in neuronal cells. Importantly, administration of roscovitine reduced the inflammatory processes evoked by Aβ in the hippocampus, leading to the significant decrease of cytokines level.

**Conclusions:**

These studies clearly show the involvement of Cdk5 in modulation of brain inflammatory response induced by Aβ and may indicate this kinase as a novel target for pharmacological intervention in AD.

**Electronic supplementary material:**

The online version of this article (10.1186/s12974-017-1027-y) contains supplementary material, which is available to authorized users.

## Background

The development of Alzheimer’s disease (AD) has been attributed to excessive accumulation of amyloid β (Aβ) and hyperphosphorylated MAP tau protein causing the formation of senile plaques and neurofibrillary tangles, respectively. Although these molecular mechanisms are recognised as fundamental to almost all aspects of AD pathogenesis [1], recently, various intracellular signaling molecules, such as cyclin-dependent kinase 5 (Cdk5, EC 2.7.11.22), glycogen synthase 3β, and mammalian target of rapamycin, have also been implicated in the development of AD [2–4]. Among these, Cdk5 has been identified as a key mediator of AD pathogenesis [5, 6].

Cdk5 belongs to the group of proline-directed serine-threonine cyclin-dependent kinases (Cdks). Contrary to cell cycle-associated Cdks, Cdk5 plays a critical role in regulation of mammalian central nervous system development, as well as synaptic plasticity and neurotransmission [7–9]. The main mechanism responsible for pathological overactivation of Cdk5, which was observed in many diseases of the central nervous system, involves calpain-mediated cleavage of activator proteins p35 and p39. Although complexes of Cdk5 with truncated peptides p25 or p29 are not catalytically more active than Cdk5-p35 or Cdk5-p39 [10], the significantly longer half-life, due to greater stability of p25 and p29, prolongs Cdk5 activation. In cellular experimental models, it has been observed that Aβ stimulates the cleavage of p35 to p25, and the inhibition of Cdk5 reduces Aβ-evoked cell death [11]. Also, recent in vivo studies have demonstrated the calpain-dependent deregulation of Cdk5 activity in a mouse model of AD [12–14]. Moreover, post mortem analysis of brain preparations of AD patients indicates an accumulation of p25 and an increase of Cdk5 activity [15, 16]. Another mechanism that may modify Cdk5 activity is post-translational modification of Cdk5, p35 or p39: phosphorylation, S-nitrosylation, sumoylation, ubiquitylation and acetylation [5, 17, 18]. It was previously demonstrated that increased activity of Cdk5/p25 may be responsible for the hyperphosphorylation of MAP tau, the destabilisation of the cytoskeleton and neuronal death [19]. The overexpression of p25 or p35 induces the phosphorylation of APP at Thr668, which significantly increases the secretion of Aβ peptides [20]. The increased activity of Cdk5 may also be responsible for the transcriptional activation of the BACE1 promoter and in consequence for enhanced amyloidogenesis [21]. Therefore, the deregulation of Cdk5 activity may be a potentially important factor in the Aβ-evoked neurotoxic cascade.

A plethora of scientific reports posits inflammation as a key component of AD pathomechanism, not only in late phase when massive neurodegeneration and cognitive impairment occur, but also in early phase of the disease, when no symptoms are present. The presence of senile plaques induces inflammatory response in AD brain, leading to activation of microglia and astrocytes and in consequence to increased production of pro-inflammatory mediators [22]. However, also monomeric and oligomeric Aβ may affect the function of glial cells within the brain. Aβ could activate microglia and astrocytes to induce the production of inflammatory cytokines, including interleukin 1 (IL-1), tumour necrosis factor α (TNF-α), IL-6, IL-8 and reactive oxygen species, that may directly damage neurons [22, 23]. Increasing evidence suggests that different cytokines, including interleukins, TNF-α and transforming growth factor beta (TGF-β) or interferon-γ (IFN-γ), actively participate in AD pathomechanism and may serve as diagnostic or therapeutic targets [24]. In addition, several epidemiological studies have indicated that a long-term use of nonsteroidal anti-inflammatory drugs (NSAIDs) may reduce the risk of developing AD [25, 26]. Some data suggest that inflammation may be an initiating factor of the cascade leading to overproduction of Aβ and to neurodegeneration [27–30]. The recent discoveries demonstrated that Cdk5 is involved in regulation of peripheral inflammatory processes, but little is known about the role of Cdk5 in regulating inflammation-related signalling in AD. It was demonstrated that chronic inflammatory reaction induces activation of Cdk5, leading to hyperphosphorylation of target proteins. In vitro studies showed that Cdk5 may be activated by interleukin-6 (IL-6) and TNF-α, and it plays an important role in inflammation-related signalling [31–33]. Moreover, our recent results indicate the activation of Cdk5 is an essential factor regulating inflammation-related gene expression in the hippocampus during systemic inflammatory response (SIR) [34]. The experiments on transgenic mouse model of AD suggested that there is interplay of inflammatory reaction with Cdk5 [35, 36]. Also, roscovitine, a potent Cdk5 inhibitor, has been found to exert anti-inflammatory actions in several in vivo models [37]. Based on previous reports, we aimed to study the involvement of Cdk5 in regulation of inflammatory signalling in the mouse brain after single intracerebroventricular (icv) injection of Aβ oligomers.

## Methods

### Materials

HFIP-pretreated amyloid β(1–42) and amyloid β(1–42) with scrambled sequence were obtained from rPeptide (Bogart, GA, USA). Anti-Cdk5, anti-p35/p25 antibodies and roscovitine were obtained from Santa Cruz Biotechnology Inc. (Santa Cruz, CA, USA). Anti-Iba1 antibody was from Abcam (Cambridge, UK). Anti-phospho-serine antibodies were from Cell Signaling Technology (Beverly, MA, USA). Anti-mouse IgG were from GE Health Care (Little Chalfont, Buckinghamshire, UK). Anti-rabbit IgG were from Sigma-Aldrich (St. Louis, MO, USA). 2′,7′-Dichlorodihydrofluorescein diacetate (DCFH-DA) was from Cayman Chemical Company (Ann Arbor, MI, USA). Fluo-4 was from Thermo Fisher Scientific (Waltham, MA, USA). Histone H1 was from Millipore (Temecula, CA, USA). Chemiluminescent reagent Clarity Western ECL Substrate was from Bio-Rad Laboratories (Hercules, CA, USA). Protein G-Dynabeads were from (Novex, Life Technologies). The High Capacity cDNA Reverse Transcription Kit, Power SYBR Green PCR Master Mix, TaqMan Gene Expression Master Mix and TaqMan Gene Expression Assays were from Applied Biosystems (Foster City, CA, USA). Cytometric Bead Arrays (CBA) flex sets were from BD Biosciences (San Jose, CA, USA). Protease inhibitor cocktail Complete was from Roche Diagnostics GmbH (Mannheim, Germany). Acrylamide, APS, chloroform, dithiothreitol, DMSO, DNAse I, isopropanol, TEMED, Tri-reagent, anti-GFAP and anti-GAPDH antibodies and all other reagents were obtained from Sigma-Aldrich (St. Louis, MO, USA).

### Preparation of Aβ oligomers

**Fig. 1:**
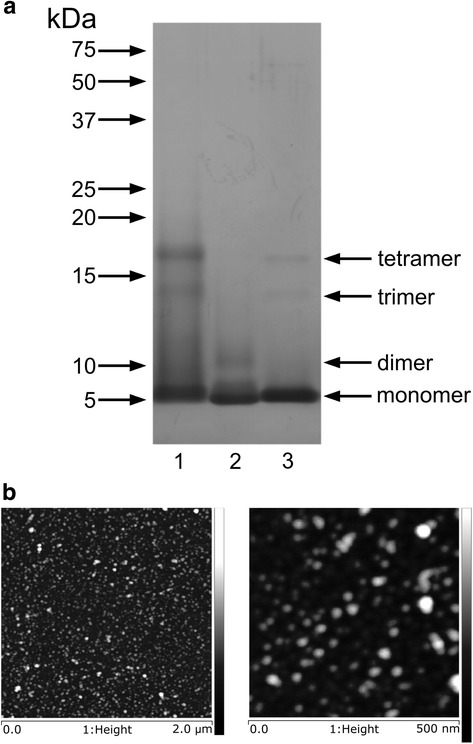
SDS-PAGE and morphological analysis of Aβ conformers. **a** Representative picture showing Aβ species after separation with 15% SDS-PAGE followed by silver staining. 1 Aβ_1–42_ after 24 h oligomerisation, 2 Aβscr after 24 h oligomerisation and 3 Aβ_1–42_ without oligomerisation. **b** Typical AFM images presenting spherical structures/oligomeric structures. Presented height scale is 5 nm

Oligomerisation of Aβ_1–42_ was performed according to Stine et al. [38]. Amyloid β was dissolved (5 mM) in anhydrous DMSO and further diluted in cell culture medium Phenol Red-free Ham’s F-12 to 100 μM concentration. After 30-s vortexing, Aβ solution was incubated at 4 °C for 24 h. In accordance with previous data, 24-h incubation of Aβ_1–42_ monomers at physiological ionic strength and neutral pH at 4 °C yielded small-size oligomeric assemblies of Aβ, mainly trimers and tetramers (Fig. 1a). Additionally, conformation state of Aβ was confirmed by using Thioflavin T (ThT) [39]. The same protocol was applied for Aβ_1–42_ with scrambled sequence of amino acids (Aβ_scr_). Freshly prepared solutions of Aβ_1–42_ were examined by atomic force microscopy (AFM) to confirm their structure. Typical result is shown in Fig. 1b. The spherical structures with a mean height of 2.5–3.5 nm determined by particle analysis method were observed. Such results are with good agreement to other works presenting low molecular oligomers [40, 41]. Aβ preparations were used directly after oligomerisation.

### Atomic force microscopy

Multimode 8 Nanoscope atomic force microscope (AFM, Bruker, USA) was used to image the surfaces of the mica substrate and the freshly deposited oligomeric structures. Silicon cantilevers, ETALON series with a spring constant of ca. 3.5 Nm^−1^ (NT-MDT, Russia) or HQ:NSC19/No Al type with a spring constant of ca. 0.5 Nm^−1^ (Mikromasch, Bulgaria) were applied for imaging in PeakForce Tapping™ Force Microscopy Mode. Calibration of the microscope was achieved by the imaging of calibration gratings supplied by the manufacturer. The images presented in this work are height type images. The examination of surfaces for artefacts by AFM, and the reproducibility, was performed in the common way, i.e. by changing the AFM cantilever and moving the sample in the *X* or *Y* direction or by varying the scanning angle and scan rate. Oligomeric samples were prepared by applying a drop of 10 μl Aβ_1–42_ solution on freshly cleaved mica (Ted Pella Inc., USA). After incubation for 10 min, the sample was rinsed with deionised water (Millipore Inc., USA) and dried under a gentle stream of argon.

### Animals

All the experiments were carried out on male C57BL/6 mice, 3 months old, supplied from the Animal House of Mossakowski Medical Research Centre PAS (Warsaw, Poland) which runs breeding of small rodents in SPF standard. The animals were maintained under controlled conditions of temperature and humidity with 12-h light/dark cycle. All of the experiments conducted on the animals were approved by the IV Local Ethics Committee for Animal Experimentation in Warsaw and were carried out in accordance with the EC Council Directive of November 24, 1986 (86/609/EEC), following the ARRIVE guidelines and guidelines published in the NIH Guide for the Care and Use of Laboratory Animals and the principles presented in the “Guidelines for the Use of Animals in Neuroscience Research” by the Society for Neuroscience. All efforts were made to minimise animal suffering and to reduce the number of animals used. Injections were performed between 9 a.m. and 1 p.m. All manipulations were performed gently and quickly to avoid stress-induced alterations.

Aβ _1–42_ was administered intracerebroventricularly (icv) at the dose of 0.5 nmol per mice as previously described by Cakala and co-workers [42]. In brief, the mice were anesthetised by intraperitoneal (ip) injection of ketamine/xylazine cocktail (100/10 mg/kg b.w.) and placed in a stereotaxic frame (Stoeling Co., USA). A 1-mm hole was drilled 1 mm posterior to the bregma and 1.3 mm lateral. A microsyringe with a 26-gauge stainless steel needle (Hamilton) was inserted to a 2-mm depth, and 5 μl of Aβ solution was slowly injected for 5 min. The control animals received injection of the solvent. Separate groups of mice received additional ip injection of potent and selective Cdk5 inhibitor, roscovitine (seliciclib, CYC202). Roscovitine was dissolved in DMSO, diluted to the desired concentration with saline and administered intraperitoneally at a dose of 50 mg/kg b.w. as described previously by Czapski and co-workers [34]. The animals from the respective experimental groups received an appropriate volume of the solvent. Roscovitine was injected directly (30 min) before injection of Aβ. The animals were then returned to their home cage. Then, after the appropriate time (3 h or 1, 3, 7, 14, 21 and 35 days) after injection, the mice were decapitated, the brains were dissected and the hippocampi were isolated on ice-cold Petri dish. The tissue was used immediately or was frozen in liquid nitrogen and stored in −80 °C until analysis. Every effort has been made to minimise the number of animals used and reduce the amount of pain, distress and/or discomfort.

### Cell culture and treatment

Human neuroblastoma SH-SY5Y cell line was obtained from Merck and was cultured in F12/MEM medium supplemented with 15% heat-inactivated foetal bovine serum (FBS), 1% non-essential amino acids, 50 units/ml penicillin and 50 μg/ml streptomycin and L-glutamine. BV2 microglia were maintained in RPMI supplemented with 5% heat-inactivated FBS, 50 units/ml penicillin and 50 μg/ml streptomycin and L-glutamine at 37 °C. Cell lines were cultured at 37 °C with 5% CO_2_ and 95% relative humidity. The cells were seeded into 60-mm and 35-mm culture dishes or 96-well plates, and the growth medium was changed into standard Hanks’ Balanced Salt Solution (HBSS). Then, the cells were treated with exogenous Aβ oligomers (10 μM) for 3 h. Suitable solvent was added to respective controls.

### Fluorometric measurements of changes in [Ca^2+^]_i_

Changes in intracellular Ca^2+^ ([Ca^2+^]_i_) concentration in SH-SY5Y and BV2 cells were monitored using the fluorescent calcium-sensitive probe Fluo-4. Its acetoxymethyl ester derivative, Fluo-4 AM, easily penetrates plasma membranes, and inside the cells, it is cleaved by esterases to Fluo-4, which becomes highly fluorescent after binding with Ca^2+^. The experiment was performed as described previously by Wilkaniec et al. [43]. SH-SY5Y and BV2 cells were seeded onto 96-well dark plates at the density of 1.4 × 10^5^ cells/ml. After 24 h, the cells were loaded with 10 μM Fluo-4 AM supplemented with 0.02% Pluronic® F-68 for 60 min at 37 °C in a HBSS. The cells were washed three times with HBSS and, to ensure complete AM ester hydrolysis, kept for 30 min at 37 °C in the dark. After a second washing, the fluorescence was measured using a microplate reader FLUOstar Omega (Ortenberg, Germany) set at 485-nm excitation and 538-nm emission wavelengths. After determining the baseline fluorescence of the cells incubated in HBSS, the changes in fluorescence after the addition of the test compounds were recorded every 15 s for 6 min. This 6-min treatment did not have any significant impact on cell viability. The results of fluorescence measurements are presented as percent changes in fluorescence intensity relative to the basal level versus duration of measurement (%F/F0). To quantify the change in the dynamics of the Ca^2+^ responses, the area under the curve (AUC) was calculated as a measure for the increase in intracellular Ca^2+^ [44].

### Measurement of intracellular free radical level

Measurement of the free radical level was carried out using fluorescent indicator 2′,7′-dichlorodihydrofluorescein diacetate (DCFH-DA) (Cayman Chemical Company), as described previously [45]. DCFH-DA is intracellularly deacetylated to 2′,7′-dichlorodihydrofluorescein (DCFH) and then oxidised to a fluorescent compound, 2′,7′-dichlorofluorescein (DCF). SH-SY5Y and BV2 cells were incubated in DCFH-DA (10 μM) solution in HBSS with 20 mM Hepes (pH 7.4) and 0.02% Pluronic for 50 min at 37 °C in the dark. Then, the cells were washed three times, and the DCF fluorescence was measured using a microplate reader FLUOstar Omega (Ortenberg, Germany) at 485-nm excitation and 538-nm emission wavelengths. After determining the baseline fluorescence of the cells incubated in HBSS, the change in fluorescence after the addition of the test compounds was recorded 3 h after treatment. The results of fluorescence measurements are presented as percent of corresponding control.

### Western blotting

Immunochemical analysis of protein level and phosphorylation was performed by Western blotting method in standard conditions. The sample was mixed with Laemmli buffer (2×) and denatured for 5 min at 95 °C. After standard SDS-PAGE on polyacrylamide gel, the proteins were transferred onto a nitrocellulose membrane and detected with specific antibodies. GAPDH level was analysed as a loading control. Densitometric analysis and size-marker-based verification was performed with TotalLab4 software.

### Analysis of the mRNA level

RNA was isolated by using TRI-reagent according to the manufacturer’s protocol (Sigma Aldrich, St. Louis, MO, USA). Digestion of DNA contamination was performed by using DNase I according to the manufacturer’s protocol (Sigma Aldrich, St. Louis, MO, USA). RNA quantity and quality were controlled by spectrophotometric analysis and gel electrophoresis. A reverse transcription was performed by using the High Capacity cDNA Reverse Transcription Kit according to the manufacturer’s protocol (Applied Biosystems, Foster City, CA, USA). Quantitative PCR was performed on an ABI PRISM 7500 apparatus using primers pair: *Nos2* forward 5′- GGCAGCCTGTGAGACCTTTG-3′ and *Nos2* reverse 5′-GCATTGGAAGTGAAGCGTTTC-3′ [46]. The levels of mRNA for *Cdk5*, *Cdk5r1*, *Il1b*, *Il6*, *Il10*, *TNF-α* and *Actb* were analysed by using the commercially available TaqMan Gene Expression Assays Mm00432437_m1, Mm00438148_m1, Mm00434228_m1, Mm00446190_m1, Mm00439614_m1, Mm00443258_m1 and ACTB_4352341E, respectively, according to the manufacturer’s instructions (Applied Biosystems). *Actb* was analysed as a reference gene. The relative levels of mRNA were calculated applying the ΔΔCt method.

### Analysis of cytokine level

The level of cytokines was determined in tissue lysates by using Cytometric Bead Array (CBA) flex sets according to the producer’s (BD Biosciences) protocol. The method is a multiplexed bead-based immunoassay that allows simultaneous measuring of the levels of multiple proteins in one sample by flow cytometry. Briefly, tissue lysates were prepared according to the producer’s protocol and stored in −80 °C until analysis. Fifty microliters of each sample or standard were mixed with all capture beads (50 μl) and incubated in the dark for 1 h at room temperature (RT). Then, the phycoerythrin (PE)-conjugated detection reagent (50 μl) was added, and the tubes were incubated in the dark for 1 h at RT. After addition of wash buffer (1 ml), the samples were centrifuged at 200×*g* for 5 min and resuspended in 300 μl of wash buffer and immediately analysed by flow cytometry. Data acquisition (300 events for each cytokine) was performed by using a BD FACSCanto II flow cytometer with BD FACSDiva Software and FCAP Array software, version 3.0 (BD Biosciences, San Jose, CA, USA). Provided standards were used to build standard curves for each cytokine. Each cytokine’s concentration was indicated by their fluorescence intensity, calculated from a standard curve and normalised to protein level.

### Cdk5 kinase assay

Activity of Cdk5 was determined with semi-quantitative method based on immunochemical measuring of phospho-serine level at recombinant histone H1 phosphorylated by immunoprecipitated Cdk5, according to Brooks [47] with modifications. SH-SY5Y cells were treated with 1–10 μM Aβ for 3 h, lysed in buffer containing 50 mM Tris-HCl (pH 7.4), 0.25 M NaCl, 0.1% *v*/*v* Nonidet P-40, 5 mM EDTA, 50 mM NaF, 1 mM Na_3_VO_4_, 1 mM Na_4_P_2_O_7_, 10 mM benzamidine, 50 μg/ml PMSF, 10 μg/ml TPCK, 10 μg/ml STI, 1 μg/ml aprotinin and 1 μg/ml leupeptin on ice. The protein extracts were combined with 25 μl Protein G-Dynabeads (Novex, Life Technologies), which were previously pre-incubated with mouse anti-Cdk5 (1 μg) antibody (Santa Cruz Biotechnology) overnight at 4 °C. Cell lysates were rotated with antibody-bound Dynabeads at 4 °C for 1 h, and the obtained complexes were sequentially washed three times with lysis buffer and then wash buffer (50 mM Tris-HCl pH 7.4, 10 mM MgCl_2_, 1 mM dithiothreitol). A kinase reaction buffer consisting of 50 mM Tris-HCl pH 7.4, 10 mM MgCl_2_, 1 mM dithiothreitol, 100 μM ATP, and 1.3 mg/ml histone H1 was added to the protein samples, followed by incubation at 23 °C for 60 min. The kinase reaction was then terminated by addition of 5× Laemmli buffer. The samples were boiled for 5 min and separated by standard SDS-PAGE, followed by transfer onto a nitrocellulose membrane and detection with anti-phospho-Ser antibody.

### Statistical analysis

All experiments were carried out at least in triplicate. The presented data are means ± SEM. The distribution of data was tested by the Anderson-Darling normality test, and parametric and non-parametric tests were used for normal and non-normal distribution, respectively. Two group comparisons were done using Student’s *t* test. Multiple comparisons were analysed by one-way analysis of variance ANOVA with Bonferroni post hoc test or with Kruskal-Wallis nonparametric test with Dunn’s multiple comparisons post hoc test. Statistical significance was accepted at *p* < 0.05. The statistical analyses were performed by using GraphPad Prism version 5.0 (GraphPad Software, San Diego, CA).

## Results

**Fig. 2:**
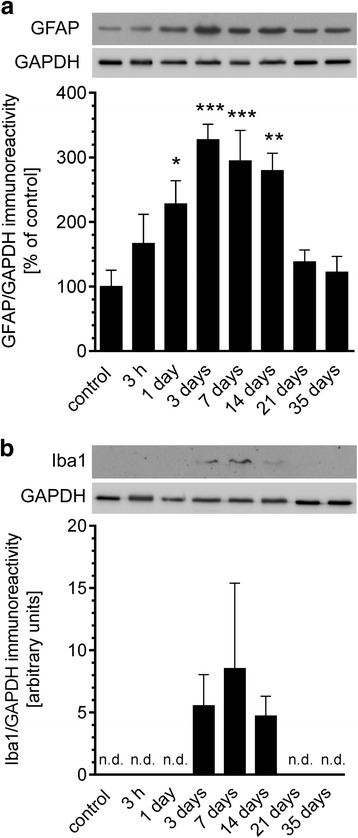
The effect of Aβ on astrocytes and glia activation in mouse hippocampus. Immunoreactivity of glial fibrillary acidic protein (GFAP) (**a**) and Iba1 (**b**) was analysed by SDS-PAGE and Western blotting in mouse hippocampus 3 h and 1, 3, 7, 14, 21 and 35 days after icv Aβ (0.5 nmol) injection. Representative pictures were shown. Results of densitometric analysis were normalised to immunoreactivity of GAPDH, as a loading control. Results are presented as the mean ± SEM from four independent experiments (*n* = 4) per time point. *p < 0.05; **p < 0.01; ***p < 0.001 versus control using a one-way ANOVA followed by the Bonferroni test. n.d. not detected

**Fig. 3:**
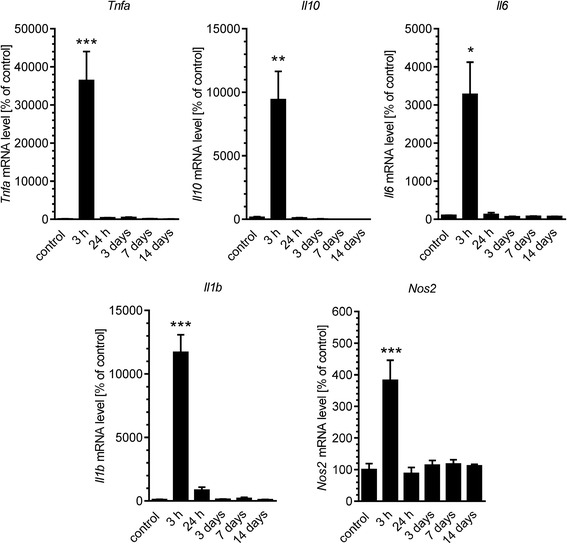
Relative changes in mRNA levels for TNF-α, IL-10, IL-6, IL-1β and iNOS in the hippocampus after Aβ treatment. Aβ (0.5 nmol) was injected intracerebroventricularly and gene expression was analysed 3 h and 1, 3, 7 and 14 days after injection by quantitative RT-PCR. Results were normalised to β-actin gene expression and are presented as the mean ± SEM from 4 to 12 independent experiments (*n* = 4–12). *, **, ***p < 0.05, 0.01 and 0.001 compared with the respective control using nonparametric Kruskal-Wallis followed by Dunn’s multiple comparisons test

**Fig. 4:**
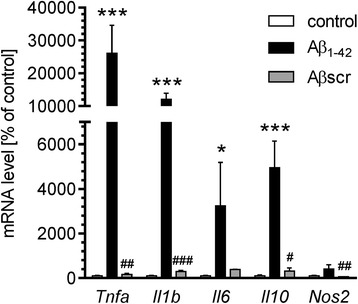
Scrambled Aβ does not change the mRNA levels for selected cytokines and iNOS in the hippocampus. Aβ (0.5 nmol) or Aβ scrambled (Aβscr, 0.5 nmol) were injected intracerebroventricularly and gene expression was analysed 3 h after injection by quantitative RT-PCR. The results were normalised to β-actin gene expression and are presented as the mean ± SEM from 6 to 12 independent experiments (*n* = 6–12). *, ***p < 0.05 and 0.001 compared with the respective control; ^#^, ^##^, ^###^*p* < 0.05, 0.01 and 0.001 compared with the Aβ_1–42_-treated animals using nonparametric Kruskal-Wallis followed by Dunn’s multiple comparisons test

To assess the overall inflammatory status of the brain, murine hippocampi were collected during a time course: 3 h and 1, 3, 7, 14, 21 and 35 days after Aβ icv injection. In these samples, the protein levels of two markers associated with the identification of microglia (ionised calcium-binding adapter molecule 1, Iba-1) or astrocytes (glial fibrillary acidic protein, GFAP) were determined (Fig. 2). GFAP level was significantly increased in the brain beginning at 24 h after injection and continued until 14 days, whereas Iba-1 immunoreactivity was modest and not detectable until 3 days after Aβ treatment. We observed the normalisation of this protein’s level 21 days after the injection. To analyse the changes in transcription of several cytokines (IL-10, IL-6, IL-1β, and TNF-α) and inducible NO synthase (Nos2), involved in inflammatory response in murine hippocampus after Aβ icv injection, we performed a real-time PCR analysis. The levels of mRNA for all examined proteins elevated significantly 3 h after Aβ injection and then returned to the basal levels at 24 h (Fig. 3). To demonstrate that the activation of inflammatory response in the brain does not depend on introduction of foreign antigen, but it is specifically induced by Aβ_1–42_ oligomers, we analysed the effect of Aβ with a scrambled amino acid sequence (Aβ_scr_) which was prepared by the same method as Aβ_1–42_ oligomers. We observed that in comparison with Aβ_1–42_, Aβ_scr_ did not exert any significant effect on gene expression of investigated proteins 3 h post-injection (Fig. 4).

**Fig. 5:**
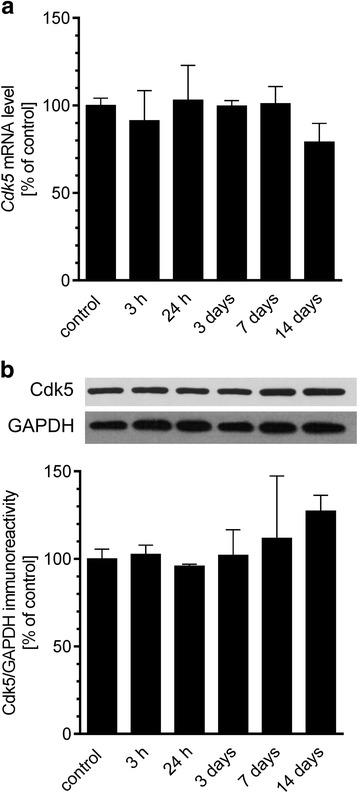
The effect of Aβ administration on Cdk5 expression and immunoreactivity in hippocampus. Aβ (0.5 nmol) was injected intracerebroventricularly. **a** Gene expression for *Cdk5* was analysed 3 h and 1, 3, 7 and 14 days after injection by quantitative RT-PCR. **b** Immunoreactivity of Cdk5 was analysed 3 h and 1, 3, 7 and 14 days after injection by SDS-PAGE and Western blotting. Representative pictures were shown. Results of densitometric analysis were normalised to immunoreactivity of GAPDH, as a loading control. Results are presented as the mean ± SEM from four independent experiments (*n* = 4)

**Fig. 6:**
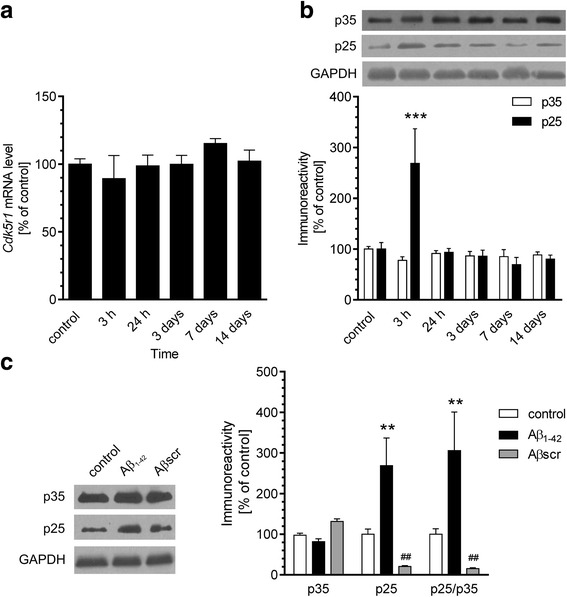
The effect of Aβ administration on p35 expression and immunoreactivity in hippocampus. Aβ (0.5 nmol) was injected intracerebroventricularly. **a** Gene expression for *Cdk5r1* was analysed 3 h and 1, 3, 7 and 14 days after injection by quantitative RT-PCR. **b** Immunoreactivity of p35 and its degradation product p25 was analysed 3 h and 1, 3, 7 and 14 days after injection by SDS-PAGE and Western blotting. Representative pictures were shown. Results of densitometric analysis were normalised to immunoreactivity of GAPDH, as a loading control. The results are presented as the mean ± SEM from four independent experiments (*n* = 4). ***p < 0.001 compared with the respective control. **c** Scrambled Aβ does not change the immunoreactivity of p35 and its degradation product p25 3 h after injection. Representative pictures were shown. Results of densitometric analysis were normalised to immunoreactivity of GAPDH, as a loading control. Results are presented as the mean ± SEM from four independent experiments (*n* = 4). **p < 0.01 compared with the respective control (solvent injected animals), ^##^*p* < 0.01 compared with the Aβ-treated animals using a one-way ANOVA followed by the Bonferroni test

Subsequently, we analysed whether exogenous Aβ affects the molecular mechanisms responsible for regulation of Cdk5 activity in mice hippocampus. Our results demonstrated that icv administration of Aβ oligomers does not influence both the mRNA and protein levels of Cdk5 (Fig. 5) as well as the expression of Cdk5 regulator, p35 (*Cdk5r1*), at any of the investigated time points (Fig. 6a). However, the protein level of the truncated form of Cdk5 activator, p25 protein, was significantly elevated 3 h after administration of Aβ and returned to basal levels at 24 h (Fig. 6b). We also observed that truncation of p35 directly depends on the effect of Aβ oligomers, since Aβ_scr_ did not exert any significant effect on the protein level of p25 (Fig. 6c).

**Fig. 7:**
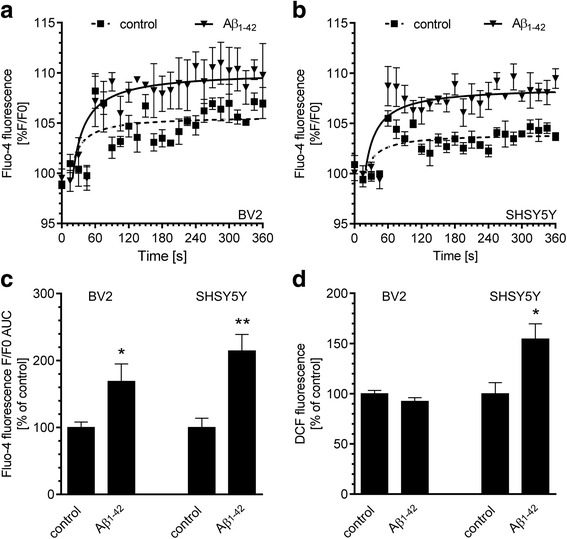
The effect of Aβ oligomers on calcium homeostasis and free radical generation in SH-SY5Y and BV2 cells. Cytoplasmic calcium level in BV2 (**a**) and SH-SY5Y (**b**) cells was measured by using Fluo-4 during 6 min after treatment with 10 μM Aβ oligomers. Data represent the mean value ± SEM for three independent experiments. **c** Responses of Fluo-4 were quantitated by measuring the area under the curve (AUC) value. Data represent the mean value ± SEM for three independent experiments. *, **p < 0.05 and 0.01 compared to control using Student’s *t* test. **d** Intracellular free radical level in SH-SY5Y and BV2 cells was measured by DCF fluorescence after 3 h incubation with 10 μM Aβ oligomers. Data represent the mean value ± SEM for four independent experiments. *p < 0.05 compared to control, using Student’s *t* test

**Fig. 8:**
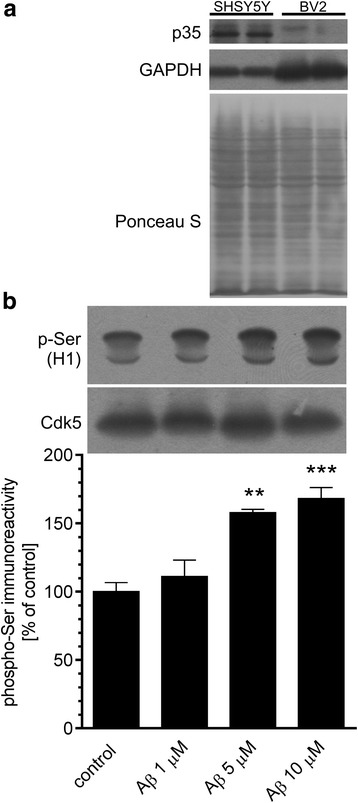
Aβ administration evokes Cdk5 activation in neuronal cells. **a** Immunoreactivity of p35 and GAPDH in SH-SY5Y and BV2 cells was analysed 3 h after Aβ treatment by SDS-PAGE and Western blotting. Representative pictures were shown. PonceauS staining was used as a loading control. **b** In SH-SY5Y cells treated with 1, 5 or 10 μM Aβ for 3 h, Cdk5 kinase activity was measured as described under “Experimental Procedures.” Results of densitometric analysis of phosphorylated histone H1 are presented as the mean ± SEM from four independent experiments (*n* = 4). **, ***p < 0.01 and 0.001 compared to control, using a one-way ANOVA followed by the Bonferroni test

To analyse whether Cdk5 activation upon Aβ treatment may depend on deregulation of calcium homeostasis and oxidative status in neuronal or glial cells, we performed in vitro studies on SH-SY5Y and BV2 cell lines. Undifferentiated SH-SY5Y cells have been widely utilised for in vitro experiments requiring neuronal-like cells [48], whereas BV2 line has been frequently used as a substitute of primary microglia due to similar antigen pattern and phagocytic and cytotoxic activity [49, 50]. For the in vitro studies, we used the treatment paradigm, which corresponds to the applied in vivo conditions: we treated the cells with exogenous Aβ at concentration of 10 μM for 3 h. By using Fluo-4, we analysed the intracellular calcium level and found that Aβ evoked a rapid enhancement of fluorescence in both BV2 (Fig. 7a) and SH-SY5Y (Fig. 7b) cells, thus indicating an increase in the cytosolic calcium level. Interestingly, the effect of Aβ treatment on [Ca^2+^]_i_ mobilisation was more pronounced in neurons than in microglia (Fig. 7c). Moreover, Aβ evoked significant enhancement of free radicals level in SH-SY5Y, but not in BV2 cells (Fig. 7d). Considering the differences in sensitivity to Aβ treatment between neuronal and glial cells, we next explored whether Aβ-evoked Cdk5 activation occurs equally within those cell types. We observed that the protein expression of Cdk5 activator p35 in BV2 cells was almost undetectable when compared to SH-SY5Y cell line (Fig. 8a), suggesting the insignificant activity of Cdk5 in glial cells. In addition, Cdk5 was immunoprecipitated to assess its activity using histone H1 as a substrate in Aβ-treated SH-SY5Y cells. As shown in Fig. 8b, Aβ treatment induced overactivation of Cdk5 kinase and the effect of Aβ was concentration dependent: while 5 and 10 μM Aβ oligomers evoked a significant increase in Cdk5 activity, Aβ at 1 μM concentration did not have any effect.

**Fig. 9:**
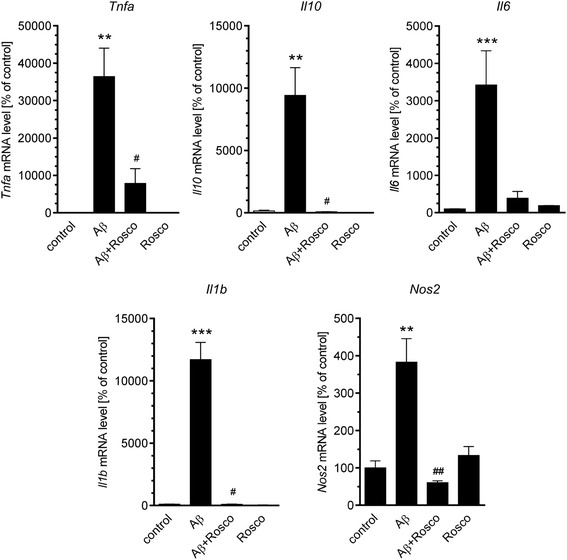
The effect of roscovitine on Aβ-induced expression of inflammation-related signalling in hippocampus. Aβ (0.5 nmol) was injected intracerebroventricularly and roscovitine (50 mg/kg b.w.) was administered intraperitoneally. Gene expression was analysed 3 h after injection by quantitative RT-PCR. Results are presented as the mean ± SEM from 3 to 12 independent experiments (*n* = 3–12). **, ***p < 0.01 and 0.001 compared with the respective control (solvent injected animals), ^#^, ^##^*p* < 0.05 and 0.01 compared with the Aβ-treated animals using nonparametric Kruskal-Wallis followed by Dunn’s multiple comparisons test

**Fig. 10:**
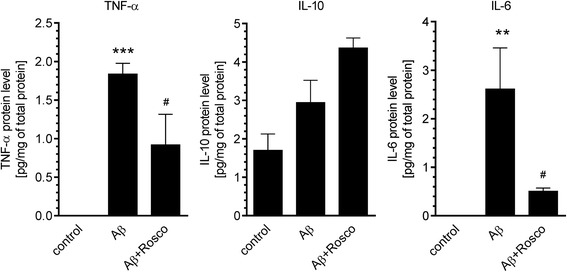
The effect of roscovitine on Aβ-induced changes in TNF-α, IL-10 or IL-6 level in hippocampus. Aβ (0.5 nmol) was injected intracerebroventricularly and roscovitine (50 mg/kg b.w.) was administered intraperitoneally. Cytokine level was analysed 3 h after injection by Cytometric Bead Array (CBA). Results are presented as the mean ± SEM from three to four independent experiments. **, ***p < 0.01 and 0.001 compared with the respective control, ^#^*p* < 0.05 compared with the Aβ-treated animals using a one-way ANOVA followed by the Bonferroni test

To investigate the role of Cdk5 in Aβ-induced inflammatory signalling in the hippocampus, the effect of Cdk5 inhibition on the expression and protein level of inflammation-related proteins was studied. We used the potent Cdk5 inhibitor, roscovitine, that was previously demonstrated to be able to inhibit Cdk5 in various animal models [6, 12, 34, 51, 52]. It was previously shown that roscovitine was able to cross blood-brain barrier and to rapidly accumulate in the brain leading to transient inhibition of Cdk5 [53–56]. As shown on Fig. 9, treatment with roscovitine (50 mg/kg b.w.) significantly decreased expression of Aβ-induced genes: *Tnfa*, *Il1b*, *Il10* and *Nos2*. We also observed the pronounced, although not significant, inhibitory effect of roscovitine on Aβ-induced elevation of *Il6* mRNA level. To determine whether roscovitine treatment influences the protein level of selected cytokines, in our animal model, we used Cytometric Bead Array (CBA) analysis. The results demonstrated that the inhibition of Cdk5 significantly prevented the Aβ-dependent elevation of the brain level of TNF-α and Il-6 in mouse hippocampus (Fig. 10). Interestingly, we observed that despite of large elevation of mRNA for IL-10 after Aβ treatment, the level of this protein was not changed, and roscovitine treatment also did not affect IL-10 concentration (Fig. 10).

Given the nuclear localisation of Cdk5 in neurons [57] and the various transcriptional regulators as Cdk5 substrates [58], it is possible that Cdk5-dependent deregulation of cytokines synthesis after Aβ treatment occurs on the transcriptional level. Therefore, we measured the effect of Aβ treatment on protein level and/or phosphorylation of various proteins that regulate gene expression, e.g. extracellular signal-regulated kinases (ERKs) as well as nuclear factor NFκB p65 subunit (RelA) and IκBα (nuclear factor of kappa light polypeptide gene enhancer in B-cells inhibitor alpha), but we did not observe any significant changes (Additional file 1: Figure S1) what excludes the involvement of these pathways in regulation of Aβ-evoked early alterations of gene expression.

## Discussion

The amyloid cascade hypothesis postulates that extracellular liberation of Aβ due to aberrant amyloid precursor protein (APP) processing plays the key role in Alzheimer’s disease (AD) pathology. Although experimental and genetic studies confirmed enhanced amyloidogenesis in AD, the amyloid theory had many critics, mainly due to the lack of a correlation between the severity of cognitive impairment and the load of senile plaques (SP) in the brain. Importantly, the attempts to develop therapeutic methods based on an anti-Aβ approach have not yielded satisfactory results [59–62]. Those observations suggest that AD pathology is more complex and involves activation of several noxious phenomena that may be connected with each other or act independently. Recent studies from preclinical and clinical studies indicate that inflammation is a powerful pathogenetic force that contributes to and drives AD pathogenesis. It was previously demonstrated that activation of microglial cells may act as a functional link between Aβ deposition and neuronal degeneration. However, the molecular mechanisms underlying Aβ induced pro-inflammatory signalling is not fully understood. In this study, we showed for the first time that activation of Cdk5 is an initiating factor of Aβ peptide-induced neuroinflammation in mouse hippocampus.

In AD brain, Aβ deposition in senile plaques is connected with prolonged and widespread activation of microglia and astrocytes [63, 64]. The majority of transgenic rodent models of AD are also characterised by extensive accumulation of activated glia and astrocytes [65, 66] long before the appearance of plaque and tangle pathology [67, 68]. Moreover, in mouse models of AD, the degree of inflammatory response correlated with development of various pathological features as well as neuronal death [67, 69]. In the present study, we used the well-characterised animal model of neurotoxicity induced by administration of Aβ_1–42_ into the lateral ventricle of C57BL/6 mice to determine the molecular mechanisms underlying Aβ peptide-induced neuroinflammation. Among several animal models that have been developed to investigate the course of AD pathology, the icv injection of amyloid peptides into the brain was observed to be appropriate for the analysis of potential mechanisms of inflammatory response, because it activates astrocytes and microglia [70–74] and produces profound neurodegeneration [70, 75–77]. Moreover, using this experimental model, the significant behavioural changes such as memory deficits as well as reduced locomotion and exploration were observed [42, 78]. Indeed, our data demonstrated that icv administration of Aβ_1–42_ resulted in the pronounced increase of the astrocytic (GFAP) and microglial (Iba-1) markers as well as induced cytokine synthesis, suggesting that this treatment significantly affects the extent of reactive gliosis. Those observations are consistent with the previous data showing that microglia and astrocytes are preferentially associated with certain amyloid plaque types [79, 80]. Amyloid peptides, their precursor protein APP and neurofibrillary tangles are potent glial activators [81, 82]. Activated microglia has a beneficial role in reducing Aβ accumulation by its phagocytosis via scavenger receptors (SRs) [83] and subsequent degradation [84]. The microglial activation is therefore a protective mechanisms promoting Aβ clearance and hinder the AD progression at the early stages of the disease. However, the persistent microglial activation stimulated by Aβ via the receptor for CD36 [85], Fc receptors, Toll-like receptors (TLRs) [86], complement and receptors for advanced glycation end-products (RAGE) [87] can increase Aβ production and decrease Aβ clearance, ultimately causing neuronal damage. Therefore, disruption of the Aβ formation delays and decreases microglial activation [88] leading to reduction of inflammatory cytokines production [89], lowering of Aβ deposition [90] and amelioration of behavioural damage [91]. Similar as microglial cells, astrocytes are known to be important for Aβ clearance and degradation, for providing trophic support to neurons and for forming a protective barrier between Aβ deposits and neurons [92]. However, astrocytes could also be a source for Aβ, because they express APP and β-secretase (BACE1), and treatment with cytokines or Aβ_1–42_ may activate amyloidogenic APP processing that drives feed-forward mechanism that promotes Aβ production in astrocytes [93]. It is well recognised that the activation of microglia and astrocytes is accompanied by increased production of pro- and anti-inflammatory cytokines, including interleukins (ILs), interferons (IFNs) and tumour necrosis factors (TNFs), as well as chemokines, nitric oxide (NO) and reactive oxygen species [94, 95]. In cultured astrocytes and microglia, Aβ treatment significantly increases the secretion of several inflammatory cytokines [80, 96] that can be significantly inhibited by the anti-inflammatory agents [80, 97]. Here, we show that both gene expression and protein level of selected pro-(IL-1β, TNF-α, IL-6) and anti-(IL-10) inflammatory cytokines as well as iNOS were increased following Aβ injection in murine hippocampus. This suggests that stimulation of cytokine release is a result of glia and astrocyte activation induced by Aβ. Interestingly, we observed that elevated cytokine expression significantly precedes increase in GFAP and Iba-1 immunoreactivity. These data are consistent with previous studies showing that increase in immunoreactivity of Iba-1 or GFAP as well as morphological alterations of microglia and astrocytes were delayed following the lipopolysaccharide (LPS) injection and corresponded with a resolution phase of microglia activation [98]. Also, in the study using LPS injection in a model of chronic neurodegeneration, differential cytokine induction in glia and astrocytes were detected independent of morphological differences [99].

As indicated above, chronic inflammation could be the consequence of AD pathology that further exacerbates the deleterious effects exerted by Aβ. However, there is still considerable debate over exactly what is the molecular mechanism of Aβ-induced neuroinflammation. The potential role of Cdk5 gained one of the highest scientific interests since it was suggested that Cdk5 may exist at a crossroad of inflammation and neurodegeneration [100–103]. In vitro studies showed that Cdk5 may be activated by pro-inflammatory mediators, and it plays an important role in inflammation-related signalling [31–33]. Previous reports showed that abnormal Cdk5 signalling is also an important component of the molecular mechanism of toxicity of AD-related proteins like Aβ, alpha-synuclein or Tau [45, 104–107]. Moreover, Cdk5 has a substantial role in either direct or indirect interactions of those proteins common to, and critical in, different neurodegenerative diseases [5]. It was demonstrated that Aβ treatment induces the conversion of p35 to p25 in primary cortical neurons, causing the prolonged activation of Cdk5 [15, 108]. The Cdk5-induced neurotoxicity after Aβ treatment was shown to be, at least in part, mediated by rapid nuclear dispersion and mislocalisation of Cdk5 in the nucleus, where it triggered the activation of several pro-apoptotic genes via activation of c-JUN pathway [109]. Cdk5 activation was also demonstrated to be responsible for Aβ-induced tau phosphorylation at Ser 396/404 in lipid rafts [110]. Consistently with the previous reports, our data showed that icv administration of Aβ induces the increased production of p25 in mouse hippocampus. Interestingly, the observed elevation of p25 level seems to be specific for neurons, as microglial cells display residual expression of p35 protein. This corresponds with the previous data showing that disruption of p35 gene significantly affects neuronal function, but other cell types are not affected and histologically normal [111]. Moreover, the expression of both Cdk5 activators p35 and p39 is restricted principally to the nervous system and their expression in microglia and astrocytes is very low [112]. Although some studies demonstrated the activation of Cdk5 in non-neuronal cells, this seems to be regulated by other proteins, e.g. cyclin D, cyclin E, cyclin I [113–115]. Therefore, in the mouse hippocampus, the Aβ-triggered activation of Cdk5 via p35/p25-dependent pathway occurs exclusively in neuronal cells. The accumulation of p25 in AD brain has been previously associated with inflammation and astrogliosis along with synaptic damage [12, 100, 102, 116]. Moreover, the overactivation of Cdk5 was previously demonstrated to induce the proinflammatory gene transcription and resulted in enhanced phosphorylation of tau and glycogen synthase kinase 3β during systemic inflammatory response [34]. In accordance with those data, we demonstrated that upon Aβ treatment, the activity of Cdk5 in neuronal cells is significantly increased as well as that Cdk5 inhibition resulted in significant reduction of mRNA level for TNF-α, IL-1β, IL-10 and iNOS in animals treated with Aβ. However, the most evident effect of roscovitine was observed for both gene expression and protein level of TNF-α and IL-6, which are two major cytokines involved in initiating and regulating the cytokine cascade during an inflammatory response but also have both direct and indirect neurotrophic effects as well as regulate cognitive function [117–120]. TNF-α release was previously shown to be involved in Aβ-induced learning and memory deficits in AD [121, 122]. Several anti-TNF-α treatments have prevented Aβ deposition, behavioural impairments and inflammation in AD animal models [36–39], suggesting that TNF-α is a detrimental factor in AD course and can serve as a reliable AD target. Similarly, IL-6 was reported to increase Aβ levels by stimulation of APP expression and processing in primary rat cortical neurons [123, 124]. IL-6 furthermore enhanced neuronal damage induced by Aβ [125]. On the other hand, overexpression of IL-6 in APP transgenic mice leads activation of microglia to a predominantly beneficial phenotype, which results in phagocytosis of Aβ but not its degradation [126]. In pathological conditions, both IL-6 and TNF-α may be also responsible for an increase in intraneuronal p35 level and Cdk5 activation [33, 101]. Interestingly, our data showed that although Aβ significantly elevates the expression for IL-10, the protein level for this anti-inflammatory cytokine is unchanged after Aβ treatment. Previous reports indicated that IL-10 production principally depends on mRNA stability and protein translation rather than the mRNA level [127], and rapid induction and degradation of IL-10 mRNA was shown to be mediated by the activation of pattern recognition—Toll-like receptors 2 (TLR2) [128]. Since TLR2 was demonstrated to be a primary receptor for Aβ to trigger neuroinflammatory activation in microglia [129], it is though possible that in our experimental model, the stimulation of the TLR2 resulted in inhibition of IL-10 translation. Although this hypothesis remains to be elucidated, still the lack of IL-10 response after Aβ treatment suggests the certain deficiency in microglia inhibition as well as failure of down-regulation of IL-1β, IL-6, TNF-α secretion, that is mainly mediated by this anti-inflammatory cytokine [130–132]. This seems to be consistent with the recent studies on several AD animal models, where overexpression of IL-10 weakened the phagocytosis of soluble Aβ by microglia and exacerbated Aβ deposits [133–135].

It was demonstrated that the molecular mechanism by which Aβ is able to stimulate cytokine production involves the nuclear factor-kappaB (NF-κB)-dependent activation of extracellular signal-regulated kinase (ERK) and mitogen-activated protein kinase (MAPK) pathways [50]. Our previous data showed that LPS-induced SIR involves Cdk5-dependent activation of NF-κB with subsequent enhancement of transcription of several proinflammatory cytokines [34]. In other studies, inhibition of Cdk5 with roscovitine decreased phosphorylation of kinase IKKβ, IκB and p65, leading to decrease of transcriptional activity of NF-κB, and, in consequence, prevented LPS-evoked expression of iNOS, COX-2, IL-1β and IL-6 [32]. For these reasons, we investigated whether Cdk5 might have a similar effect on Aβ-mediated activation of NF-κB in mouse hippocampus. Surprisingly, we did not observe any changes in NF-κB–ERK–MAPK pathway after Aβ treatment. Similarly, we did not detect any alteration in phosphorylation of glycogen synthase kinase 3β and transcription factor MEF2. Since our study demonstrated that glial cells are less sensitive to Aβ treatment and the abundance of Cdk5 co-activator, p35 protein is greatly reduced in those cells; thus, it is possible that the Aβ-induced p25 formation and Cdk5 activation in neurons may indirectly influence the glial cells to produce cytokines. While it was demonstrated that neurons can release cytokines and chemokines as well as respond to them by way of cytokine and chemokine receptors [136], the direct involvement of neurons in the inflammatory response is marginal when compared to activated glial cells that are the major source of inflammatory mediators in the brain [137]. In response to injury, neurons could produce adhesion molecules and trophic factors that recruit and activate microglial cells and astrocytes [136]. Previous reports demonstrated that p25 overexpression in neurons resulted in upregulation of cytosolic phospholipase A2 (cPLA2) and lysophosphatidylcholine (LPC) release, by which it activated the surrounding microglia and astrocytes [102]. Moreover, LPC was shown to be a potent chemoattractant for T cells [138, 139], and p25 overexpression was demonstrated to initiate the peripheral cell recruitment into the brain to exacerbate neuroinflammation [102]. Therefore, it is also possible that LPC release due to p25 formation in neurons might be also responsible for the peripheral cell recruitment. While this interesting hypothesis remains to be further elucidated in our experimental conditions, it is still most probable that Aβ-induced p25 overexpression in neurons activates the surrounding microglia, and the subsequent cytokine release is a consequence rather than primary trigger originating from p25-expressing neurons [100]. Since we have observed that cytokine expression subsided and even disappeared in time after Aβ injection, we take this as corroborating the hypothesis that Cdk5 activation in neurons leads to secretion of extracellular factors, such as LPC, that stimulate microglia, and the levels of those factors decrease in the brain as fewer neurons remain to produce them.

## Conclusions

Summarising, our results clearly indicate the important role of Cdk5 in regulating early inflammation-related gene expression induced by Aβ. These might suggest Cdk5 inhibition as novel potential therapeutic targets for inhibition of brain inflammation during AD.

## Additional file

Additional file 1: Figure S1. The effect of Aβ administration on immunoreactivity of proteins. Aβ (0.5 nmol) was injected intracerebroventricularly. Immunoreactivity of analysed proteins was examined by SDS-PAGE and Western blotting 3 h after injection of Aβ. Results of densitometric analysis were normalised to immunoreactivity of GAPDH, as a loading control. The results are presented as the mean ± SEM from four independent experiments (*n* = 4). (TIFF 499 kb)

## Abbreviations

AD: Alzheimer’s disease
Aβ: Amyloid beta
CBA: Cytometric bead array
Cdk5: Cyclin-dependent kinase 5
COX-2: Cyclooxygenase 2
cPLA2: Cytosolic phospholipase A2
EDTA: Ethylenediaminetetraacetic acid
ERK: Extracellular signal-regulated kinase
GAPDH: Glyceraldehyde 3-phosphate dehydrogenase
GFAP: Glial fibrillary acidic protein
HBSS: Hanks’ Balanced Salt Solution
Iba-1: Ionised calcium-binding adapter molecule 1
IFN-γ: Interferon gamma
IL-1: Interleukin
iNOS: Inducible nitric oxide synthase
LPC: Lysophosphatidylcholine
MAP: Microtubule associated protein
MAPK: Mitogen-activated protein kinase
MEF2: Myocyte enhancer factor-2
NaF: Sodium fluoride
PE: Phycoerythrin
PMSF: Phenylmethylsulfonyl fluoride
RAGE: Receptors for advanced glycation end products
RT: Room temperature
SIR: Systemic inflammatory response
STI: Soybean trypsin inhibitor
TGF-β: Transforming growth factor beta
TLR2: Toll-like receptor 2
TNF-α: Tumour necrosis factor alpha
TPCK: Tosyl phenylalanyl chloromethyl ketone
